# Characterization of non‐IgA vasculitis: Demographic, clinical, and treatment‐related features in a retrospective analysis of 28 biopsy‐confirmed cases from a German university hospital

**DOI:** 10.1111/1346-8138.17545

**Published:** 2024-11-18

**Authors:** Inga Hansen‐Abeck, Alessandra Rünger, Lisa Piepke, Julian Kött, Anna Giordano‐Rosenbaum, Anne Menz, Finn Abeck, Stefan W. Schneider

**Affiliations:** ^1^ Department of Dermatology and Venereology University Medical Center Hamburg‐Eppendorf Hamburg Germany; ^2^ Institute of Pathology University Medical Center Hamburg‐Eppendorf Hamburg Germany

**Keywords:** cutaneous leukocytoclastic vasculitis, cutaneous vasculitis, small‐vessel vasculitis

## Abstract

Non‐IgA vasculitis is a rare disease that belongs to the group of small‐vessel vasculitides. Due to nomenclature and classification changes introduced in 2018, there are few published data under this name. The aim of this study is to characterize non‐IgA vasculitis as an independent vasculitis entity in terms of demographic, clinical, and treatment‐related features. A retrospective data analysis of patients with biopsy‐confirmed non‐IgA vasculitis treated at the Department of Dermatology at the University Medical Center Hamburg‐Eppendorf between January 1, 2018, and December 31, 2022, was performed. A total of 28 patients with non‐IgA vasculitis were included; 53.6% (15/28) were women and 42.9% (12/28) were older than 71 years. Previous infection as a possible triggering factor was found in 42.6% (12/28) of the cases. Palpable purpura was the most common skin finding (78.6%, 22/28) and 28.6% patients (8/28) had skin lesions above the waist. On direct immunofluorescence, C3 (89.3%, 25/28) was the most frequent deposition, followed by fibrinogen (71.4%, 20/28) and IgM (53.6%, 15/28). Hospitalization was required in 85.7% (24/28), with a mean hospital stay of 9.4 ± 4.1 days. No fatal courses were reported. This study is the first characterization of non‐IgA vasculitis based on patient cases from Germany and contributes to a better understanding of non‐IgA vasculitis as an independent entity. Non‐IgA vasculitis primarily affects older patients of both sexes, with most cases having an identifiable trigger. Our results indicate that cutaneous manifestations often extend beyond the lower legs. Treatment is usually required in the inpatient setting and requires a longer stay than other dermatological conditions. With proper treatment, the disease is not expected to be fatal.

## 1 INTRODUCTION

Inflammation of the blood vessels can occur in any organ but is particularly common in the skin.^1^ Cutaneous vasculitis is a heterogeneous group of diseases that can be divided into three groups based on skin involvement: a cutaneous manifestation of systemic vasculitis, a purely cutaneous variant of systemic vasculitis, and as cutaneous single‐organ vasculitis.^1^, ^2^ Systemic vasculitis involves at least one other organ beyond the skin, whereas cutaneous single‐organ vasculitis remains confined to the skin and does not progress into a systemic vasculitis.^1^ Other classifications are based on the size of the affected vessels involved.^3^ Certain types of efflorescences and their patterns of distribution are distinctive in specific vasculitis conditions, providing diagnostic clues or serving as diagnostic criteria.^3^

Immune complex vasculitis belongs to the small‐vessel vasculitis group, primarily affects the postcapillary venules, and presents clinically with palpable purpura, most frequently on the lower extremities.^1^, ^4^ The group of immune complex vasculitides can be further classified into cryoglobulinemic vasculitis, urticarial vasculitis, and hypocomplementemic vasculitis, as well as IgA and non‐IgA vasculitis.^1^, ^4^ Non‐IgA vasculitis (also known as IgM/IgG vasculitis) was listed as a separate entity for the first time in the 2018 update of the Chapel Hill Consensus Conference (CHCC) nomenclature of vasculitides and assigned to the group of ‘single‐organ vasculitides’.^1^ Histopathologically, non‐IgA vasculitis presents as leukocytoclastic vasculitis of the small vessel, especially prominent in the postcapillary venules, while direct immunofluorescence (DIF) typically shows a deposition of IgM or complement factors, with IgG deposition being less frequent.^5^, ^6^

Prior to diagnosing non‐IgA vasculitis, other immune complex vasculitis subtypes should be excluded.^5^ However, specific diagnostic criteria for non‐IgA vasculitis do not yet exist,^1^, ^4^ thus complicating the diagnostic process, especially since histological differentiation from IgA vasculitis is not necessary in the absence of clinical symptoms such as abdominal pain, joint pain, abnormal urine examination, and presentation above the waistline.^7^

In the past, non‐IgA vasculitis has not been included in the group of small‐vessel vasculitis, nor was it described as leukocytoclastic vasculitis.^1^, ^8^ Leukocytoclasia, however, is a description of the disintegration of neutrophil granulocyte nuclei that is typical of vasculitis.^9^ This histopathological feature is neither specific for non‐IgA vasculitis nor pathognomonic for vasculitis, and, therefore, equating the diagnosis of non‐IgA vasculitis with leukocytoclastic vasculitis is misleading.^3^ Due to the complex nomenclature and classification, limited data have been published under the terms non‐IgA vasculitis and IgG/IgM vasculitis. It is likely that these data were published prior to the 2018 CHCC classification as hypersensitivity vasculitis or leukocytoclastic vasculitis and therefore cannot be clearly distinguished from other subtypes. Previous categorization efforts by Loricera et al. described the group of cutaneous single‐organ vasculitis of small vessels after the publication of the revised nomenclature for vasculitis in 2012, but this classification is now also outdated.^2^

Non‐IgA vasculitis is a rare disease with an estimated annual incidence of approximately 48 per 1 million, and, not surprisingly, published literature on non‐IgA vasculitis and IgM/IgG vasculitis is scarce.^10^ A retrospective characterization from Italy of 41 patients revealed a female patient majority as well as lesions predominantly localized to the lower leg.^11^ As most of the demographic and clinical features of non‐IgA vasculitis are unknown,^4^ this study aims to characterize non‐IgA vasculitis as a distinct entity according to the CHCC nomenclature of vasculitides in terms of demographic, clinical, and treatment‐related features based on biopsy‐confirmed patient cases treated at the University Medical Center Hamburg‐Eppendorf.

## 2 METHODS

**FIGURE 1:**
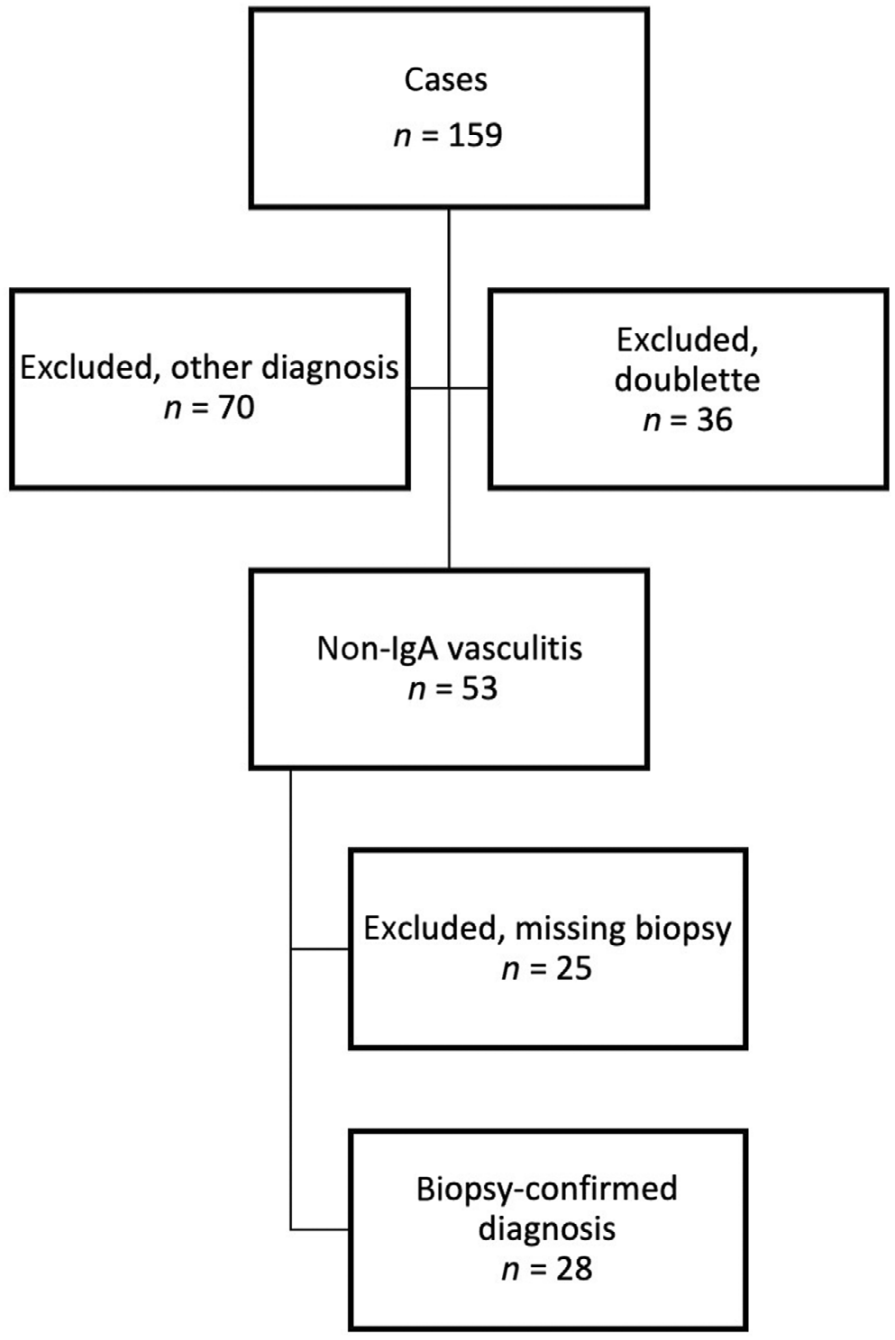
Flow chart of inclusion and exclusion criteria of patients included in the analysis.

A retrospective data analysis of patients with non‐IgA vasculitis who were treated at the Department of Dermatology and Venereology at the University Medical Center Hamburg‐Eppendorf between January 1, 2018, and December 31, 2022, was performed. The patients were identified using the *International Statistical Classification of Diseases and Related Health Problems* (*ICD‐10*) codes M31.0 (hypersensitivity angiitis), L95.9 (vasculitis limited to skin, unspecified), and D69.0 (allergic purpura). The search yielded 159 patient cases, of which 28 patients met the inclusion and exclusion criteria (Figure 1).

All patients with non‐IgA vasculitis, defined as a confirmed acute leukocytoclastic small‐vessel vasculitis that did not meet clinical, laboratory, or histological diagnostic criteria for other small‐vessel vasculitides, were included. The following prespecified inclusion and exclusion criteria were applied:

- Inclusion criteria:
  - Presence of acute leukocytoclastic small‐vessel vasculitis on histological examination
  - Absence of IgA deposition in DIF
- Exclusion criteria:
  - Diagnosis of other vasculitis (e.g. IgA vasculitis, ANCA‐positive vasculitis)
  - Histology and/or DIF were not performed
  - Systemic involvement

The following data were recorded from the electronic patient records:

- Demographic: sex, age, and health insurance
- Clinical: body mass index, Charlson Comorbidity Index (CCI; a scoring system that allows prediction of long‐term mortality based on specific diseases and patient age^12^), symptoms and duration of symptoms, triggering factors, pretreatment, cutaneous efflorescence and localization, blood cell count, retention and inflammatory parameters, urinalysis, histology, and DIF
- Treatment‐related factors: therapy, type, and duration of treatment

Data analyses were performed using GraphPad Prism software version 8 (GraphPad Software). Descriptive statistics are summarized as mean ± standard deviation.

The study was conducted in accordance with the 1964 Declaration of Helsinki. The study design was reviewed and approved by the ethics committee of the Hamburg Medical Association prior to commencement of the study (reference number: 022‐100 973‐BO‐ff).

## 3 RESULTS

In total, 28 patients with non‐IgA vasculitis were identified. A total of 53.6% (15/28) of patients were female, and the mean age was 58.9 ± 21.0 years (range, 21–85 years) (Table 1). Most patients (42.9%; 12/28) were 71 years or older.

**TABLE 1:** General patient characteristics of study patients with non‐IgA vasculitis.

Variable	Patients (*n* = 28)
Age	
Mean ± SD	58.9 ± 21.0
Range	21–85
Age group	
≤30 years	4 (12.3)
31–50 years	6 (21.4)
51–70 years	6 (21.4)
≥71 years	12 (42.9)
Sex, *n* (%)	
Male	13 (46.4)
Female	15 (53.6)
BMI	*n* = 26
Mean ± SD	28.9 ± 6.7
Range	20.0–50.0
CCI	
Mean ± SD	2.4 ± 2.1
Range	0–6
Health insurance, *n* (%)	
Public	26 (92.6)
Private	2 (7.4)
Treatment setting	
Outpatient	4 (14.3)
Inpatient	24 (85.7)

Abbreviations: BMI, body mass index; CCI, Charleson Comorbidity Index; SD, standard deviation.

On initial presentation to our clinic, patients reported a mean duration of cutaneous changes of 12.2 ± 11.5 days (Table 2). Symptoms included cutaneous pain in 42.9% (12/28), pruritus in 21.4% (6/28), and joint pain in the ankle or knee in 14.3% (4/28). Neither ankle nor knee pain was classified as systemic involvement.

**TABLE 2:** Clinical characteristics of study patients with non‐IgA vasculitis.

Variable	Patients (*n* = 28)
Duration of cutaneous symptoms at first visit (in days)	
Mean ± SD	12.2 ± 11.5
Range	1–44
History of vasculitis, *n* (%)	6 (21.4)
Pretreatment	8 (28.6)
Symptoms (multiple answers), *n* (%)	
Skin pain	12 (42.9)
Pruritus	6 (21.4)
Joint pain	4 (14.3)
Fever	2 (7.1)
Efflorescences (multiple answers), *n* (%)	
Palpable purpura	22 (78.6)
Maculae	12 (42.9)
Papules	8 (28.6)
Necrosis	4 (14.3)
Hemorrhages	2 (7.1)
Ulcerations	2 (7.1)
Erosions	1 (3.6)
Localization, *n* (%)	
Below the waist	20 (71.4)
Lower legs	11 (39.2)
Legs and thighs	6 (21.4)
Up to the abdomen	2 (7.1)
Above the waist	8 (28.6)
Trunk and upper extremity	6 (21.4)
Trunk	2 (7.1)

Abbreviation: SD, standard deviation.

**FIGURE 2:**
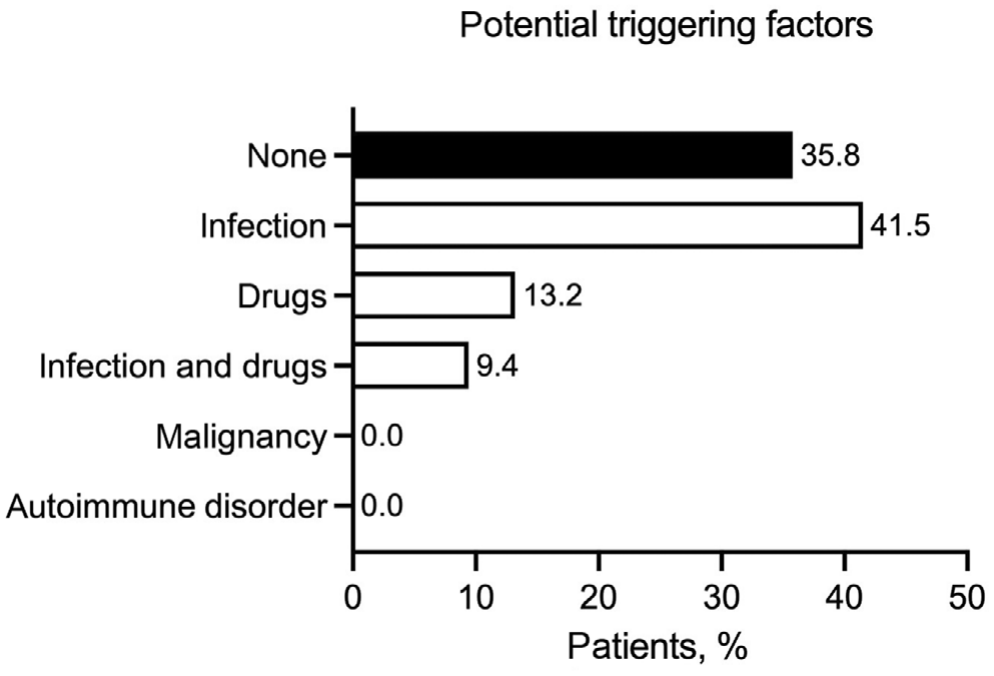
Potential trigger factors.

Regarding possible triggering factors, 42.6% (12/28) of patients reported a previous infection in the past 4 weeks (Figure 2). With regard to previous infections, seven patients reported infections of the respiratory tract, three patients reported urinary tract infections, and two patients reported soft tissue infections. Pathogen diagnosis of the previous infection was not routinely performed because, in most cases, the infection had resolved by the time patients presented to our department. In addition, six patients reported that they had previously taken a medication (four nonsteroidal anti‐inflammatory drugs, one amoxicillin, and one infliximab). Further studies should include a detailed infection workup. A total of 21.4% (6/28) of patients had a recurrence of previously known non‐IgA vasculitis and 28.6% (8/28; mostly with oral prednisolone) had received previous outpatient treatment.

**FIGURE 3:**
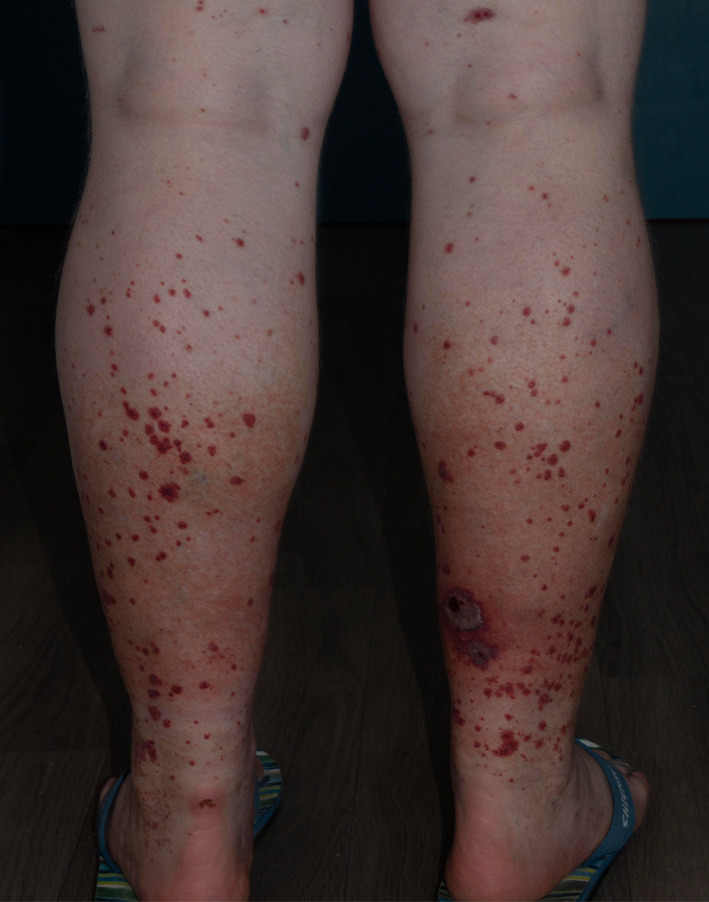
Clinical image: dermatological features of non‐IgA vasculitis. Patients often present with palpable purpura on the lower legs. The picture shows a relatively severe case of non‐IgA vasculitis with ulcerations.

In terms of skin lesions, 78.6% (22/28) of patients had palpable purpura, 14.3% (4/28) had necrosis, and 7.1% (2/28) had ulceration (Figure 3). In 39.2% (11/28) of patients, the skin lesions were limited to the lower legs, and in 28.6% (8/28) the lesions extended above the waist (Table 2).

Laboratory tests showed normal blood cell counts in 32.1% (9/28) of patients and a normal C‐reactive protein in 35.7% (10/28). Leukocytosis was found in 17.9% (5/28) of the patients, but three of the five patients had previously received systemic steroid therapy.

**FIGURE 4:**
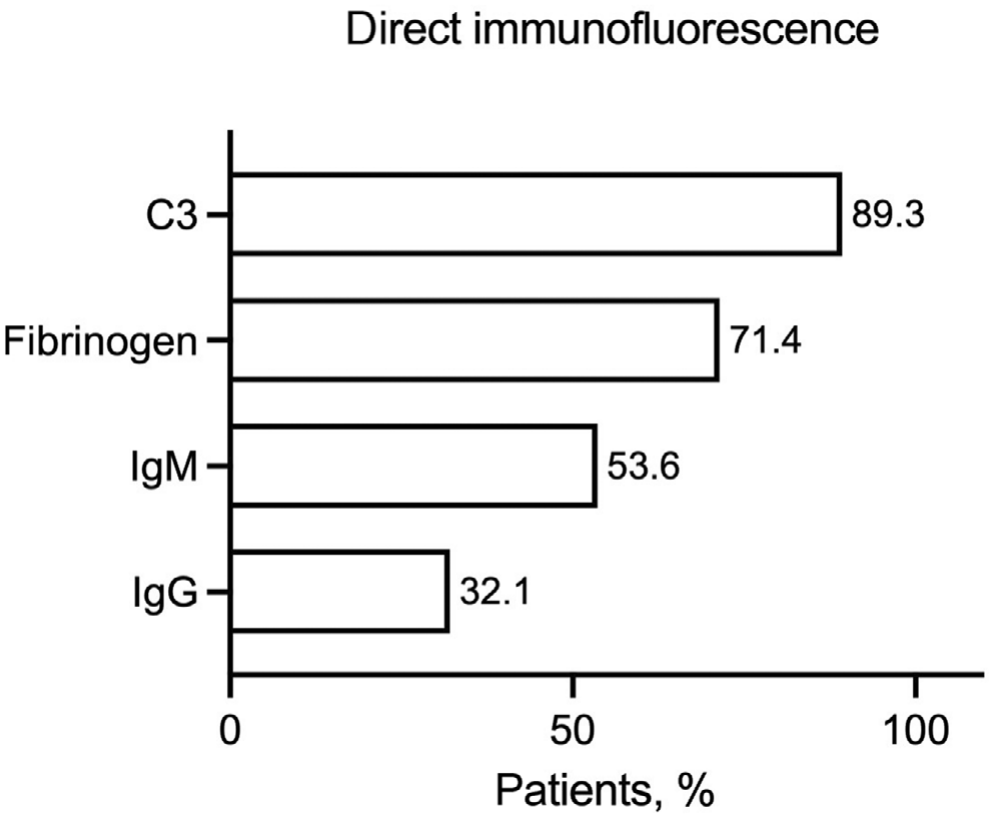
Distribution of the fluorescence pattern in direct immunofluorescence.

**FIGURE 5:**
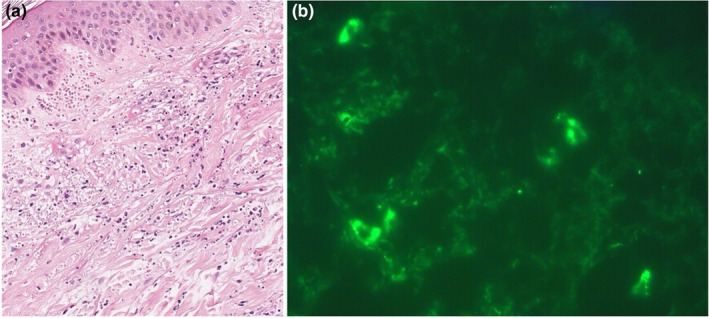
Histologic and direct immunofluorescence features. (a) Histologic image showing leukocytoclastic vasculitis with dermal perivascular infiltrates of neutrophils and leukocytoclasia (hematoxylin–eosin staining, original magnification ×20). (b) Direct immunofluorescence demonstrating granular deposition of C3 in the superficial dermal vessel walls (original magnification ×63).

All patients underwent biopsy with histological examination and DIF. All patients revealed histological evidence of acute leukocytoclastic small‐vessel vasculitis (100%, 28/28). DIF showed the most frequent deposition of C3 (89.3%, 25/28), followed by fibrinogen (71.4%, 20/28), IgM (53.6%, 15/28), and IgG (32.1%, 9/28) (Figures 4 and 5).

A urine test was performed in 92.9% (26/28) of the patients and showed abnormalities in 61.5% (16/26), although these were mostly mild (single positive). The creatinine‐albumin ratio was determined in nine patients and showed an abnormal value in 44.4% (4/9), with a range of 59.4 to 269.2, whereupon a nephrological consultation was performed to rule out renal involvement.

Inpatient treatment was required in 85.7% (24/28) of cases. The average length of hospital stay was 9.4 ± 4.1 days (range, 1–23 days; one discharge against medical advice after 1 day).

All patients received systemic therapy; 50% (14/28) received oral prednisolone and 50% (14/28) received intravenous prednisolone (Table 3). Steroid‐sparing immunosuppressants were used in 7.2% (2/28) due to lack of improvement or relapse. Local therapy included topical glucocorticoids in 75.0% (21/28) of patients and compression therapy in 78.6% (22/28).

**TABLE 3:** Treatment‐related characteristics.

Variable	Patients (*n* = 28)
Systemic therapy (multiple answers possible), *n* (%)	
Prednisolone	28 (100.0)
Intravenous	14 (50.0)
Oral	14 (50.0)
Oral dapsone	1 (3.6)
Oral azathioprine	1 (3.6)
Local therapy (multiple answers), *n* (%)	
Compression therapy	22 (78.6)
Topical steroids	21 (75.0)
Wound therapy	4 (14.3)
Duration of inpatient stay (in days)	*n* = 24
Mean ± SD	9.6 ± 4.1
Range	1–23
Fatal course, *n* (%)	0 (0)

Abbreviation: SD, standard deviation.

A total of 91.6% (22/24) of inpatients were discharged home, with two cases requiring transfer to another department, but not because of vasculitis complications. A total of 7.1% (2/28) of patients returned to the clinic because of relapse.

## 4 DISCUSSION

Non‐IgA vasculitis is a poorly described entity of small‐vessel vasculitis that was first described as an independent disease in the CHCC vasculitis nomenclature in 2018.^1^, ^4^ We present data from 28 biopsy‐confirmed patients who were treated for non‐IgA vasculitis in our clinic over the past 5 years. The aim of this study was to characterize patients with non‐IgA vasculitis with regard to demographic, clinical, and treatment‐related features. To our knowledge, there is only one comparable study from Italy that characterized patients with cutaneous IgM/IgG vasculitis.^11^

The mean age of patients in our study population was 58.9 years, with an almost balanced sex ratio. In comparison, the mean age of patients in the study by Marzano et al. was 50 years, with 61% being female.^11^ Although non‐IgA vasculitis can occur at any age (the youngest patient in our study population was 21 years), the disease seems to affect older patients more frequently. In our study population, 42.9% of patients were older than 70 years. The mean CCI was 2.4 ± 2.1, which is comparable to the median CCI, which was assessed in a cohort of patients with IgA vasculitis.^13^

Palpable purpura, previously described as a clinically characteristic sign of small‐vessel vasculitis, was the most common skin finding in our cohort. However, the exclusive involvement of the lower legs has previously been described as typical of non‐IgA vasculitis; this was also shown in the cohort of Marzano et al.^3^, ^11^ In contrast, we found that only 39.2% of cases had skin manifestations limited to the lower legs, while 28.6% had skin lesions above the waist. Neither localization nor histology (hematoxylin‐eosin staining) can reliably distinguish non‐IgA vasculitis from IgA vasculitis; therefore, direct immunofluorescence is required.^1^, ^11^ A possible explanation for the high proportion of patients with lesions above the waist could be that they sought treatment at the university clinic. Consequently, half of the patients received intravenous prednisolone therapy, as this form of administration allows for higher doses and a prompt response requested in those patients (the maximum rate in our sample was 1.7 mg/kg body weight). Assuming that patients with mild forms of vasculitis are not primarily treated in university centers, the severity of non‐IgA vasculitis in our patient population may be overestimated. Consistent with this assumption, a higher case mix index, which indicates the average severity of patient cases, was found in university hospitals compared with nonuniversity hospitals.^14^ Further data collection is needed to determine whether this is an exception or whether the severity of non‐IgA vasculitis has been underestimated to date.

We found a recurrence rate of non‐IgA vasculitis of only 7.1%, which is much lower than the rate reported by Marzano et al. (24.4%). Because of the retrospective nature of our study, patient follow‐up was not possible, and thus presentation in another hospital due to relapse cannot be excluded. Despite lacking knowledge of the exact frequency and risk factors, disease relapse remains important.

For most cases, possible vasculitis trigger factors were identified, with infections being the most common. An association with infections and drugs has been previously described in vasculitis; however, from our data, it is not possible to assess whether a causal relationship exists.^6^ In the CHCC nomenclature, these cases are grouped under *vasculitis of probable etiology*.^1^, ^4^

On DIF, C3 was most frequently detected in our cohort, followed by fibrinogen, IgM, and IgG. Similar distributions have been reported in the literature, although fibrinogen was not investigated in all studies.^15^, ^16^, ^17^ However, some of these previous studies were published before the introduction of the CHCC 2018 nomenclature and refer to leukocytoclastic vasculitis without further differentiation. In contrast to the work by Marzano et al. (characterization of IgM/IgG vasculitis), our study also included patients without deposition of IgG or IgM, provided that no IgA was detectable, and we therefore use the term non‐IgA vasculitis instead of IgG/IgM vasculitis in our study. We cannot exclude that our cohort includes patients in whom IgA or IgM/IgG was already depleted, which could occur because of inappropriate sampling location for skin biopsy. However, due to our clinic's focus on vascular diseases, we consider the possibility of an inappropriate sampling location to be low.

Until now, non‐IgA vasculitis has been considered as a mild form of small‐vessel vasculitis. We found that the patients who received inpatient treatment had a mean length of stay of 9.6 ± 4.1 days, with a range of 1 to 23 days (one discharged against medical advice). For all dermatoses with inpatient treatment, the average length of hospital stay in Germany is 5.69 days.^14^ Assuming a correlation between disease severity and length of hospital stay, non‐IgA vasculitis requires longer treatment than the average dermatological disease, although comparability is severely limited.

Our data show that the severity of non‐IgA vasculitis may have been underestimated in the past. However, compared with other small‐vessel vasculitides (e.g. ANCA‐associated vasculitides), the course of non‐IgA vasculitis appears to be relatively benign. For example, a Colombian cohort of 106 patients (of these, 52% had granulomatosis with polyangiitis) reported a mean hospital stay of 16.6 ± 12.2 days and a mortality rate of 16.5%.^18^ There were no fatal cases in our cohort, highlighting the benign nature of the disease even in cases with extensive skin involvement. Another difference is that non‐IgA vasculitis preferentially, and almost exclusively, affects the skin.^11^ In comparison, only 18.8% of cases of ANCA‐associated vasculitis involve the skin, while manifestations in the kidneys (84.0%) and lungs (43.3%) are more common.^18^ Renal and pulmonary involvement appear to be an important factor in the higher mortality rate.^19^ In our study, the presence of ANCAs was considered an exclusion criteria. It seems unlikely that patients with ANCA‐negative ANCA‐associated vasculitis were part of our patient cohort because none of the patients showed symptoms in the ear, nose, and throat area and symptoms of systemic vasculitis such as renal (e.g. rapid progressive renal insufficiency) or pulmonary (e.g. dyspnea, cough, or hemoptysis) involvement.^20^

In addition to the retrospective study design, our data are limited by the small patient cohort. By excluding all patients whose diagnosis was based solely on clinical findings or incomplete histological diagnosis, the patient number in this study was significantly reduced. Due to the largely unexplored nature of non‐IgA vasculitis, this approach was necessary to generate valid scientific data, even though the diagnosis is often made in clinical practice without histological examination, as precise diagnostic criteria are still lacking. With this study, we aimed to characterize non‐IgA vasculitis, a disease about which much remains unknown, and further studies with larger patient populations are needed to identify clinically relevant correlations.

## 5 CONCLUSION

Due to the nomenclature introduced in 2018, there are few published data on non‐IgA vasculitis under this name. To our knowledge, this study is the first characterization of non‐IgA vasculitis based on patient cases from a German university hospital. According to our study, non‐IgA vasculitis mainly affects older patients without a gender preference, and a possible trigger factor could be identified in the majority of cases. Our results suggest that the cutaneous manifestation is often not limited to the lower legs. A variety of different fluorescence patterns are possible in DIF, while treatment is usually in‐patient and exceeds the average length of stay compared with other dermatological conditions. With adequate treatment, the disease is not expected to be fatal. Additional studies with larger patient populations are needed to further investigate this disease.

## Funding Information

This work was supported by the grant from the Deutsche Forschungsgemeinschaft (German Research Council) SCHN474/10‐1

## CONFLICT OF INTEREST STATEMENT

The authors declare no conflicts of interest.

## ETHICS STATEMENT

Approval of the research protocol was obtained by an institutional review board. The study was conducted in accordance with the 1964 Declaration of Helsinki. The study design was reviewed and approved by the ethics committee of the Hamburg Medical Association prior to commencement of the study (reference number: 022‐100 973‐BO‐ff).
